# A qualitative patient interview study to understand the experience of patients with nonalcoholic steatohepatitis

**DOI:** 10.1097/HC9.0000000000000036

**Published:** 2023-02-09

**Authors:** Mark G. Swain, Billie Pettersson, Oren Meyers, Meredith Venerus, Jan Oscarsson

**Affiliations:** 1 Liver Unit, Department of Medicine, Cumming School of Medicine, University of Calgary, Calgary, Alberta, Canada; 2 Patient Centered Science, Cardiovascular, Renal and Metabolism (CVRM), BioPharmaceuticals Medical, AstraZeneca, Gothenburg, Sweden; 3 Patient Centered Endpoints, IQVIA, New York, New York, USA; 4 Patient Centered Solutions, IQVIA, Madrid, Spain; 5 Late-stage Development, Cardiovascular, Renal and Metabolism (CVRM), BioPharmaceuticals R&D, AstraZeneca, Gothenburg, Sweden

## Abstract

NASH is a potentially progressive form of NAFLD characterized by hepatocyte injury and liver inflammation which can cause fibrosis. Currently, there are limited data on the patient experience of NASH. Our aim was to use both literature review and patient interviews to understand the signs/symptoms and life impacts of NASH fibrosis stages F1–F4 that are important to patients, as well as begin to investigate the applicability of an instrument (ie, questionnaire) that may be used to capture patients’ experiences. The literature review identified concepts (signs/symptoms and impacts) related to NASH fibrosis stages F1–F4 and the NASH-specific patient-reported outcome instrument (NASH-CHECK) for reporting patient experience of NASH. Interviews with 22 patients from Canada and the USA with NASH fibrosis stages F1–F4 revealed 27 signs/symptoms and 32 impacts that they felt were important, including fatigue, pain in the abdomen, worry, and frustration. Three concepts reported during patient interviews were not identified in the literature review. No concepts appeared to be exclusive to a specific fibrosis stage or presence/absence of obesity and no linear trends were identified between fibrosis stage or presence/absence of obesity and level of disturbance reported for concepts. The patient interviews supported the concepts included in the NASH-CHECK overall, demonstrating that it could be used to report the patient experience of NASH fibrosis stages F1–F4. Interviews with patients with NASH fibrosis stages F1–F4 revealed patients can self-report and elaborate on signs/symptoms and impacts related to the disease regardless of fibrosis stage. The NASH-CHECK was identified as a suitable instrument that could be used by patients with fibrosis stages F1–F4.

## Graphical abstract

**Figure:**
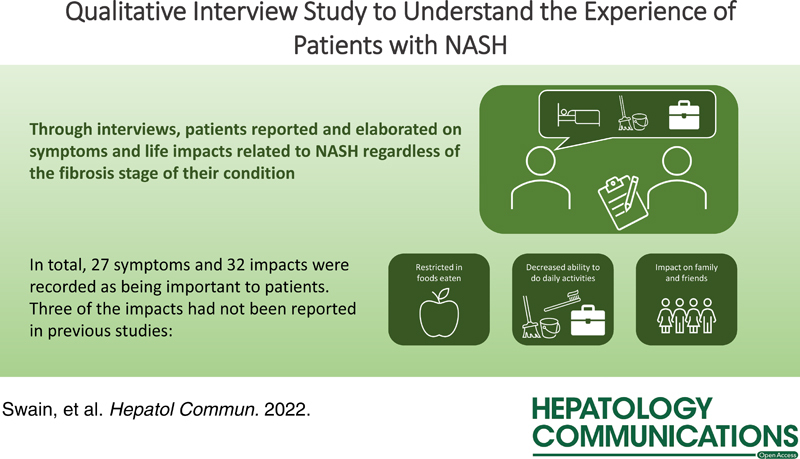

NASH is a chronic and potentially progressive liver disease and a severe form of NAFLD. The condition is estimated to affect 1.5%–6.5% of the global population.^1^ In addition to liver steatosis, NASH is characterized by hepatocyte injury and liver inflammation, which can cause fibrosis and progress to cirrhosis and HCC.^2^ Fibrosis in the context of NASH ranges from FO (no fibrosis) to F4 (cirrhosis); cirrhosis is associated with portal hypertension and increased risk of liver-related events such as ascites, variceal bleeding, hepatic encephalopathy, hepatorenal syndrome, and liver failure. NASH with advanced liver fibrosis can also increase the risk for cardiovascular disease and chronic kidney disease, and death.^3^–^7^

A number of comorbidities associated with the development of NAFLD also increase the likelihood of progressive fibrosis in those with NASH, including type 2 diabetes, obesity, and high blood pressure.^8^–^10^ Although there are therapies available to manage some comorbidities associated with NASH, there are currently no approved pharmacological treatments for the condition.^11^

NASH has traditionally been considered an asymptomatic disease, making diagnosis and monitoring challenging. Clinicians typically rely on histological diagnosis from invasive liver biopsies to diagnose patients with NASH. Although histological indicators of disease severity are important markers of NASH and risk of progression, they do not measure the impacts of the disease, or of treatments, that may be most important to patients. Understanding how symptoms and the life impacts of NASH affect patients’ well-being is increasingly recognized as important for individuals to make more informed decisions about their care, as well as for designing endpoints that measure changes in patients’ health-related quality of life in clinical trials of treatment or studies of the personal impact of NASH progression. Patients with NASH may report a variety of symptoms and disease-related impacts on their lives, including physical and psychological issues or social difficulties that negatively affect their quality of life.^12^–^15^ Improving communication between health care professionals and patients about their fibrosis stage and its associated risks, particularly for patients with moderate or severe obesity, has been found to positively correlate with better adherence of patients to lifestyle changes designed to reverse disease progression of NAFLD/NASH.^16^

Patient-reported outcomes (PROs) can be used as an important tool to document patients’ experiences of diseases or treatments, directly from the patients and without interpretation by clinicians or other individuals.^17^ Moreover, data from PROs are important for supporting medication labeling claims, health care policy, and clinical decision-making.^18^

PRO instruments can be developed to record and quantify responses from patients. By asking patients about their medical condition experience, it is possible to create and improve PRO instruments so that their content is most applicable and relevant to patients. Qualitative patient interviews can be used to develop and establish the content validity of PRO instruments.^19^

The overall goal of this study was to explore and document the concepts (signs/symptoms and impacts) that are relevant and important to patients with NASH fibrosis stages F1–F4.

## PATIENTS AND METHODS

### Study design

**FIGURE 1:**
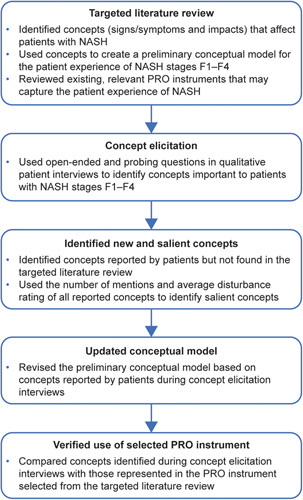
Flow chart summarizing the study. Abbreviation: PRO, patient-reported outcome.

A targeted literature review (TLR) was performed to identify concepts relevant to patients with NASH fibrosis stages F1–F4 and to identify potential PRO instruments that may be used to capture their experiences of NASH. A preliminary conceptual model was created of the signs/symptoms (signs, observable manifestations of the disease that are reported through patient expression rather than clinical examination; symptoms, internal experiences resulting from the disease of interest or comorbidities that only a patient can report) and impacts (effects of the symptoms experienced by the patients) identified as most relevant to patients with NASH fibrosis stages F1–F4. Qualitative concept elicitation interviews were conducted with patients with NASH fibrosis stages F1–F4, and the findings used to update the conceptual model. The concepts identified during the patient interviews were also compared with the PRO instrument identified during the TLR to establish whether it is appropriate for patients with NASH fibrosis stages F1–F4. An overview of the study design is in Figure 1.

### TLR and preliminary conceptual model

A TLR was performed to provide a deeper understanding of the concepts underlying patients’ experiences of NASH and explore any existing, relevant instruments that attempt to capture these experiences. The targeted search was executed using PubMed to identify literature on the signs, symptoms, and impacts of NASH. The search criteria used included the search terms “nonalcoholic steatohepatitis” and “nonalcoholic fatty liver disease” for disease, as well as “signs,” “symptoms,” “patient-reported outcomes,” and “conceptual model” for outcomes of interest (Supporting Table S1, http://links.lww.com/HC9/A89). The searches were restricted to English language publications and articles published between April 2, 2009, and April 2, 2019. The article abstracts initially identified were screened against the exclusion criteria given in Supporting Table S2 (http://links.lww.com/HC9/A89). Blog searches were also conducted on the websites PatientsLikeMe, Fatty Liver Foundation, Health Unlocked, and American Liver Foundation in which the patient clearly stated a diagnosis of NASH or NAFLD. A preliminary conceptual model of the experience of patients with NASH was created based on the concepts identified through the TLR. Concepts were grouped into “signs and symptoms,” “immediate impacts,” and “general impacts.”

During the TLR, PRO instruments were also reviewed to understand what disease-specific instruments, as well as generic instruments with a history of use in patients with NASH, existed. The PRO instrument that was considered most suitable in clinical practice and trials of NASH was selected for further investigation.

### Patient recruitment

Patients were recruited through a clinical study team at the University of Calgary, Calgary, Alberta, Canada, or with assistance from Global Perspectives—Patient Search & Engagement, Norwich, England. Patients were recruited via telephone calls by the site coordinator or clinicians as well as through clinician referral by Global Perspectives. Patients completed an informed consent form, and their physicians completed the confirmation of diagnosis form. Eligible patients were from the USA or Canada, aged over 18 years, and had a confirmed NASH fibrosis diagnosis (either by liver biopsy for stages F1–F3 or by biopsy or clinical assessment using noninvasive tests for stage 4). Patients with NASH fibrosis stages F1–F3 had to have been receiving stable treatments that could affect NASH for a minimum of 12 months before biopsy if treatment had been initiated. A summary of patient eligibility criteria and any treatment taken for comorbid conditions is given in Supporting Tables S3 and S4 (http://links.lww.com/HC9/A89). Patients received an honorarium upon completion of the interview process.

### Ethics

Ethics approval was obtained from the relevant ethics committee/institutional review board; New England Institutional Review Board (approval no. 120190204; initial approval given August 2019; now WIRB-Copernicus Group Institutional Review Board) and University of Calgary’s Conjoint Health Research Ethics Board (approval no. REB19-1306). This study was conducted in accordance with the ethical principles of The Declaration of Helsinki and is consistent with Good Clinical Practice and the regulations of the United States Food and Drug Administration, applicable laws, and the institutional review board requirements.

### Concept elicitation interviews

Concept elicitation interviews were conducted by 2 interviewers (Meredith Venerus, BSc, female, consultant, IQVIA; Stella Karantzoulis, PhD, female, principal, IQVIA) experienced in performing concept elicitation interviews. The interview approach was consistent with recommended guidelines provided by the International Society for Pharmacoeconomics and Outcomes Research Good Research Practices Task Force.^20^ Patients were interviewed one-to-one by telephone with the interviewer and, with the patients’ permission, the interviews were audio recorded. Interviewers followed an institutional review board approved (New England Institutional Review Board and University of Calgary’s Conjoint Health Research Ethics Board) semistructured interview guide and asked patients open-ended questions to allow them to spontaneously discuss the signs/symptoms and impacts of NASH that affect their lives. The interviews lasted ∼60–90 minutes. Concepts listed in the preliminary conceptual model from the TLR, and any additional concepts identified in the PRO instrument(s) as most suitable for patients with NASH from the TLR, were used to inform the study materials for concept elicitation, including acting as a starting list of probes for interviewers. During the interviews patients were also asked to rate the level of disturbance the reported concepts have on their daily lives, using a numeric scale from 0 to 10 (0 being “not disturbing at all” and 10 being “extremely disturbing”). Once patient interviews were completed, the audio files were transcribed verbatim for data analysis.

### Qualitative data analysis

Deidentified transcripts were generated from all recorded interviews. Coding of the audio transcripts was performed by Rakshit Patil and Meredith Venerus of IQVIA, who followed coding rules that were established before coding began. One primary coder was responsible for coding all interviews and a second coder coded the interview transcripts until an intercoder agreement of 0.7 or more was achieved in at least 3 transcripts.^21^ Coding was completed in the order that the interviews were conducted and transcripts were coded using ATLAS.ti, version 8 (Chicago, IL, USA) software. Concepts were categorized into “signs and symptoms” and “impacts” that affect the daily lives of patients with NASH. The frequencies and levels of disturbance for the concepts mentioned were recorded, as well as whether they were mentioned spontaneously (“spontaneous”) or required further questions by the interviewer (“probed”). Salient concepts were identified as those that were mentioned by at least 50% of patients and had a mean disturbance rating of 5 or higher (on a 0–10 scale). Concept saturation was identified by splitting the patient interviews into waves (5 interviews per wave, except for the final wave which consisted of the remaining interviews) and identifying the point at which no unique concepts were provided by the individual groups of interviewees. The qualitative focus of this study and small sample sizes meant statistical significance tests were not used to determine variations between or within populations.

### Updated conceptual model

The preliminary conceptual model created from the TLR was updated based on patient responses. New concepts identified as salient were added to the conceptual model. Concepts from the preliminary conceptual model reported by <20% of patients were removed.

### Applicability of existing PRO instrument(s)

The applicability of existing PRO instrument(s) identified during the TLR was also explored. The content validity of PRO instruments was evaluated to assess whether there is existing evidence to support their use in the NASH population. The concepts identified as salient from the concept elicitation interviews were compared with items present in the PRO instrument to confirm the suitability of the instrument for patients with NASH fibrosis stages F1–F4.

## RESULTS

### TLR

Following an initial search, 596 article abstracts (full-text publications and congress abstracts) were identified for possible inclusion; after review, 12 articles were identified as highly relevant to the patient experience of NASH/NAFLD (Supporting Table S5, http://links.lww.com/HC9/A89). This was supplemented with 8 congress posters presented at the 2019 European Association for the Study of the Liver conference (Supporting Table S5, http://links.lww.com/HC9/A89). In addition, 22 patient forum/blog posts were identified for inclusion. A total of 40 concepts relevant to NASH were selected from the articles and congress presentations; 22 symptoms and 18 impacts.

The most commonly reported symptom was fatigue (reported by 67%–78% of patients)^22^,^23^ followed by pain in the abdomen/upper right quadrant (25%–61%),^22^,^23^ unspecified bodily pain (20%–60%),^24^,^25^ and joint pain (60%).^25^ Cognitive problems (30%–57%)^23^,^24^ and abdominal bloating (35%–56%)^22^,^25^ were also frequently reported. The most commonly reported impacts included some degree of worry (26%–68%)^22^,^25^ followed by poor sleep quality (38%–56%),^22^,^25^ decreases in physical activity (issues walking upstairs or walking several blocks, (13%–56%),^24^,^25^ and limitations/frustrations associated with diet (48%–52%).^22^,^25^ Using patient blogs, 4 additional symptoms were identified that were mentioned by 3 or more blogs (low energy, dizziness, ascites/ascites leaks, and bleeding). A draft conceptual model to illustrate the signs/symptoms and impacts that affect patients with NASH was created using the concepts identified during the TLR.

Of the 14 PRO instruments reviewed, 3 were developed specifically for NASH. The NASH-specific PRO instrument (NASH-CHECK) and chronic liver disease questionnaire-NASH/NAFLD (CLDQ-NAFLD/NASH) were identified as potentially the most suitable for patients with NASH fibrosis stages F1–F4. The NASH-CHECK,^14^ which was developed by the Liver Investigation: Testing Marker Utility in Steatohepatitis (LITMUS) group, was then identified as the most suitable PRO instrument currently available for NASH in clinical practice and trials. This was in part decided because, in comparison with the CLDQ-NAFLD/NASH, the development of the NASH-CHECK followed the principles outlined in the United States Food and Drug Administration guidance for PRO instruments, and was in line with the European Medicines Agency’s reflection paper on health-related quality of life measures.^19^,^26^ In addition, following concept elicitation interviews, 2 rounds of cognitive debriefing interviews were performed to identify patient-perceived impacts of NASH for the generation of items for the NASH-CHECK instrument and to ensure patients found the final version of the instrument relevant, understandable, and clear.^14^,^27^

### Demographics

A total of 22 patients across liver fibrosis stages F1–F4 [F1, 6 (27.3%); F2, 4 (18.2%); F3, 7 (31.8%); and F4, 5 (22.7%)] were interviewed in this study (Table 1). Patients had a mean age of 51.5 years (range, 21–72 y); the majority of patients were female (63.6%), and most patients were White non-Hispanic (77.3%). The most frequently reported comorbidities were obesity (63.6%), diabetes (59.1%), and hypertension (45.5%).

**TABLE 1:** Demographic and clinical characteristics of interviewed patients with NASH

Demographic characteristics	Patients (N = 22) [n (%)]
Age (y)	
Mean	51.5
Range (minimim–maximum)	21–72
Sex	
Female	14 (63.6)
Fibrosis stage	
F1	6 (27.3)
F2	4 (18.2)
F3	7 (31.8)
F4	5 (22.7)
Ethnicity	
White non-Hispanic	17 (77.3)
Asian	1 (4.5)
Hispanic	2 (9.1)
Not specified	2 (9.1)
Geography	
Canada	11 (50.0)
USA	11 (50.0)
Comorbidities	
Asthma	2 (9.1)
Chronic diarrhea	2 (9.1)
CKD	1 (4.5)
Depression	2 (9.1)
Diabetes	13 (59.1)
Dyslipidemia	4 (18.2)
Hypertension	10 (45.5)
GERD	3 (13.6)
Obesity	14 (63.6)
Sleep apnea	4 (18.2)

Abbreviations: CKD, chronic kidney disease; GERD, gastroesophageal reflux disease.

### Concept elicitation

The concept elicitation interviews identified 59 concepts (27 signs/symptoms and 32 impacts), including 3 that were not identified during the TLR (restricted with regards to food eaten, decreased ability to do daily activities, and impact on family/friends). Most signs/symptoms and impacts were mentioned when the interviewers asked follow-up questions rather than spontaneously by the patients. Of the reported concepts, 32.2% of signs/symptoms and 26.5% of impacts were mentioned spontaneously. The 22 interviews were grouped chronologically into 5 waves (4 waves contained 5 patients; 1 wave contained 2 patients) to identify the saturation of concepts. The majority of signs/symptoms (88%) and impacts (91%) were reported in the first wave of interviews. Saturation was achieved after the second and third waves for signs/symptoms and impacts, respectively, supporting that few or no new concepts would have been identified through additional interviews.

**FIGURE 2:**
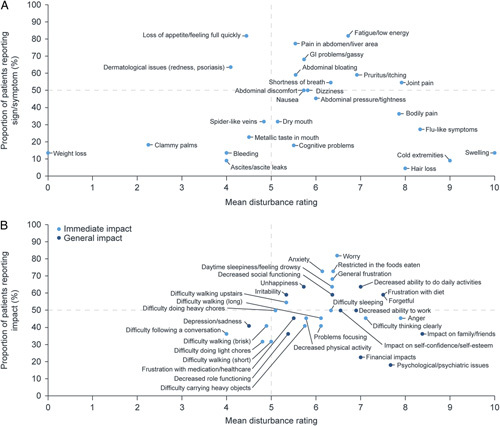
Saliency of signs/symptoms (A) and impacts reported by patients (B). Abbreviation: GI, gastrointestinal.

Ten signs/symptoms were identified as salient, with the most salient including fatigue/low energy, pain in the abdomen/liver area, and gastrointestinal problems/gassy (Figure 2A). Sixteen impacts were identified as salient, with the most salient including worry, anxiety, restricted in foods eaten, general frustration, decreased ability to do daily activities, and daytime sleepiness/feeling drowsy (Figure 2B). Of the reported impacts, 19 were reported as immediate impacts that were closely related to symptoms associated with NASH and 13 as general impacts which were considered more distal consequences of NASH symptoms or immediate impacts. A selection of patient quotes describing some of the most frequently reported salient concepts is given in Supporting Table S6 (http://links.lww.com/HC9/A89). Of the reported signs/symptoms, 4 (cognitive problems, swelling, cold extremities, and hair loss) had average disturbance ratings between 7.7 and 10 but were reported by <15% of patients. Although loss of appetite/feeling full quickly and dermatological issues were frequently reported by patients (81.8% and 63.6%, respectively), they had average disturbance scores of <5 (4.4 and 4.1, respectively). For impacts, one concept (psychological/psychiatric issues) was given a score >5 (5.5) but was reported by <20% of patients.

### Concept analysis by fibrosis stage

Of the reported signs/symptoms and impacts, none were exclusive to a specific fibrosis stage of NASH, and patients across all NASH fibrosis stages required probing for over half of their reported concepts. The average number of signs/symptoms reported by patients with NASH fibrosis stage F1, F2, F3, and F4 was 9.5, 8.0, 11.0, and 12.2, respectively. The average number of impacts reported for patients with NASH fibrosis stage F1, F2, F3, and F4 was 16.3, 8.0, 17.9, and 19.4, respectively. The small size of the populations meant differences in salience are only suggestive of differences between fibrosis stages. The reported concepts suggest some were salient only for patients with fibrosis stages F1–F3 or F4 (Table 2). Two signs/symptoms (nausea and abdominal pressure/tightness) were reported as salient only for patients with NASH fibrosis stage F4 (ie, cirrhosis). Five signs/symptoms (including gastrointestinal problems/gassy, loss of appetite/feeling full quickly, and abdominal bloating) were considered salient in patients with NASH fibrosis stages F1–F3 but not those with stage F4. Twelve impacts, which were predominantly those related to physical ability, were salient in patients with NASH fibrosis stage F4 but not those with stages F1–F3. Four impacts (including worry, frustration with diet, and unhappiness) were reported as salient for patients with fibrosis stages F1–F3 but not those with F4.

**TABLE 2:** Concepts reported as salient by interviewed patients with liver fibrosis stages F1–F3 or F4

	Fibrosis stage	
	F1–F3 (n = 17)	F4 (n = 5)
Signs/symptoms	GI problems/gassy	Abdominal pressure/tightness
	Dermatological issues	Nausea
	Loss of appetite/feeling full quickly	
	Abdominal bloating	
	Dizziness	
Impacts	Worry	Difficulty sleeping
	Frustration with diet	Difficulty thinking clearly
	Daytime sleepiness/feeling drowsy	Difficulty carrying heavy objects
	Unhappiness	Difficulty doing light chores
		Difficulty walking upstairs
		Decreased physical activity
		Difficulty walking (short)
		Anger
		Difficulty walking (long)
		Frustration with medication/health care
		Decreased role functioning
		Difficulty walking (brisk)

Abbreviation: GI, gastrointestinal.

### Obese versus nonobese populations

Over half the patient population were obese (body mass index ≥30 kg/m^2^). Although obese and nonobese patients reported similar concepts, patients who were obese reported some concepts as more disturbing than did nonobese patients, leading to the salience of certain concepts for obese patients but not for nonobese patients with NASH. The small size of the populations in this study means differences in salience are only suggestive that there are differences between obese and nonobese populations. Obese patients reported 7 signs/symptoms and 15 impacts as salient that nonobese patients did not (Table 3).

**TABLE 3:** Concepts reported as salient by only obese patients interviewed

	Obese patients (N = 14)
Signs/symptoms	Nausea
	Shortness of breath
	Dizziness
	Joint pain
	Abdominal pressure/tightness
	Abdominal discomfort
	Bodily pain
Impacts	Worry
	Frustration with diet
	Anxiety
	Daytime sleepiness/feeling drowsy
	Difficulty walking upstairs
	Impact on self-confidence/self-esteem
	Problems focusing
	Decreased physical activity
	Decreased ability to work
	Difficulty doing heavy chores
	Anger
	Difficulty walking (long)
	Difficulty carrying heavy objects
	Difficulty thinking clearly
	Frustration with medication/health care

### Conceptual model of patient experience of NASH

**FIGURE 3:**
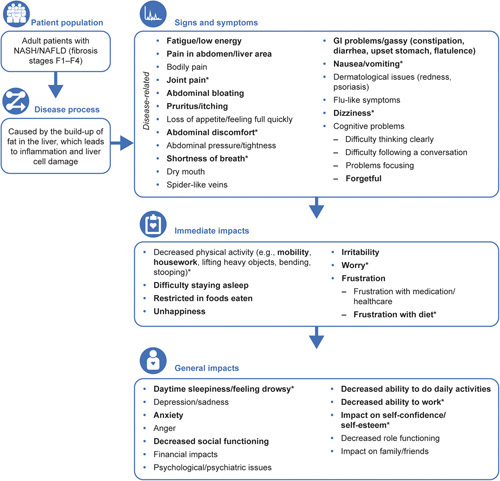
Conceptual model of signs/symptoms and impacts identified in patients with NASH stages F1–F4 from patient interviews. Bold text indicates salient concepts reported by patients. *Salient concepts that may have been driven by obese patients. Abbreviation: GI, gastrointestinal.

The preliminary conceptual model was updated based upon the concepts identified during concept elicitation interviews (Figure 3). Three impacts were added to the conceptual model as well as 4 signs/symptoms and 4 impacts were removed. Several concepts were also updated to be more representative of patients’ experience based on their responses during the concept elicitation interviews. Although psychological/psychiatric issues and cognitive problems were reported by <20% of patients, these concepts were not removed from the conceptual model. This was because the average disturbance scores were quite high and concepts associated with these, including forgetfulness and problems focussing, were reported by over 20% of patients. In addition, although some concepts were salient only in obese individuals, they were also reported in nonobese patients so were kept in the conceptual model because they are still relevant to the entire population of patients with NASH. A summary of the updates made to the conceptual model is given in Supporting Table S7 (http://links.lww.com/HC9/A89).

### Applicability of existing PRO instrument(s)

Salient concepts identified during concept elicitation were compared with items from the NASH-CHECK, which was selected during the TLR as the most appropriate PRO instrument for assessing the patients’ experiences of NASH. Similar concepts reported during the patient interviews were grouped together for comparison with items in the NASH-CHECK. Eighteen of the salient concepts (72.0%; 5/10 signs/symptoms; 13/15 impacts) were covered by the NASH-CHECK either directly or indirectly (Supporting Table S8, http://links.lww.com/HC9/A89). The salient signs/symptoms not included in the NASH-CHECK were dizziness, gastrointestinal problems/gassy (constipation, diarrhea, upset stomach, flatulence), joint pain, nausea/vomiting, and shortness of breath, and the impacts not included in the NASH-CHECK were frustration and irritability.

## DISCUSSION

There is currently limited literature on the patients’ experiences of living with NASH; traditionally the focus has often been on the diseased organ, with the impact of the disease on the diagnosed individual being perceived as subjective and hard to quantify. However, there is increasing evidence showing the range and burden of symptoms and impacts related to NASH and how they affect patients.^12^–^15^,^28^ Actively engaging patients with lived experiences of health conditions can allow more patient-centered support to be provided, including more suitable treatments, policies, and management strategies, which together can improve patient outcomes.^29^ This study supports that NASH is not an asymptomatic condition, regardless of fibrosis stage. Understanding patient experience of diseases or treatments, and not purely relying on physiological data, is important because it can help improve clinical effectiveness and patient safety such as by improving adherence to treatments.^19^,^30^

In this study, 3 impacts were identified during patient interviews that were not included in the preliminary conceptual model which was developed following the TLR. The identification of new concepts reported by patients with NASH demonstrates the importance of talking directly with patients to comprehensively capture their experience or perspective of a disease. Awareness of concepts reported by patients, in particular salient concepts, can help health care professionals to improve their knowledge of the disease, allowing them to ask more relevant questions and provide suitable support. Patients may feel more positive and supported by having impacts of the disease, beyond the known clinical symptoms, acknowledged. Improvements in understanding how to tailor treatment could also provide economic benefits—later-stage disease treatment is more costly than early stage, so a reduction in patients progressing through to late-stage disease can also be financially more favorable.^31^ Understanding the complete patient experience allows for the formation of multidisciplinary approaches to address the “whole” patient. Identifying parallels between reported signs/symptoms and impacts and fibrosis stage could also be used to monitor disease progression. For example, the identification of nausea and abdominal pressure as salient only in patients with fibrosis stage F4 suggests these symptoms are more bothersome in later stages of NASH even though they could occur in earlier stages. Although decompensated cirrhosis was an exclusion criterion, this could be due to ascites developing in cirrhotic patients.^32^ However, the small size of this patient group means significance cannot be drawn in this study and conclusions should be viewed as preliminary.

The large proportion of patients who required probing questions to report concepts that affect them may be representative of a lack of patient awareness of the signs/symptoms and impacts related to NASH, rather than representing the level of importance they hold to the patients. It could also be a consequence of patients feeling a negative societal perception of NASH, with it often being viewed as a self-inflicted condition, causing patients to be reluctant to vocalize the negative effects they experience as a result of NASH.^13^ Stigma against NASH can come from various avenues (including the general public, health care professionals, and policymakers) and can result in patients not seeking necessary health care for their condition as well as result in a reduction in the allocation of resources for the condition, both of which can lead to poor clinical outcomes.^33^ It is important to acknowledge and address the issues associated with the stigmatization of NASH, including by improving education about the condition.^33^ Patient support groups can also be beneficial to patients with health conditions such as NASH. By sharing personal experiences of NASH with other patients, and receiving some educational support from health care professionals, patients may understand their condition better as well as learn strategies for managing their condition better.^29^

It is important to recognize the concepts that were reported by few patients but received high disturbance scores [such as (signs/symptoms) cognitive problems, swelling, cold extremities, and hair loss, and (impacts) psychological/psychiatric issues]. Differences in concept disturbance between patients may indicate variation in individuals with NASH and how they experience the condition.

Some of the concepts reported in this study may not have been the direct result of NASH itself but instead caused or influenced by other comorbidities. Symptoms reported as highly disturbing by only a few patients in this study, such as swelling, cold extremities, and hair loss, are also recognized in patients with hypothyroidism and so could be indicative of this condition—NAFLD has been found to be prevalent in patients with clinical hypothyroidism and could potentially directly or indirectly impact the pathogenesis of NAFLD and NASH.^34^ Impacts such as cognitive problems are also commonly associated with chronic conditions, including obesity, diabetes, and coronary heart disease, which are also frequently associated with NASH.^35^–^39^ Diabetes has previously been associated with concepts identified in this study, including mental impacts of the condition, joint pain, and challenges associated with changing the types of food patients are able to eat or activities they are advised to undertake.^40^,^41^ Obesity is often associated with tiredness, joint pain, low physical stamina, and depression.^42^ Fitness levels of patients could also have influenced the types of concepts and their level of “disturbance” reported; lower levels of fitness could cause more negative reporting of impacts related to physical activities or deconditioning. Such factors could affect patients with later-stage NASH, in particular, especially those with cirrhosis because patients with later-stage NASH have previously been found to have worse overall health and lower fitness levels than those with earlier fibrosis stages.^43^ Some patients within this study also had comorbid gastrointestinal conditions (including gastroesophageal reflux disease, and ulcerative colitis) which could result in similar signs/symptoms to those reported for NASH.^44^,^45^ Similarities between NASH and potential comorbid conditions support that further research into how concepts reported by patients align with NASH or comorbid conditions is required.

Of the impacts reported, decreased social functioning was identified as a salient impact irrespective of fibrosis stage or whether patients were obese. Loneliness frequently occurs in patients with chronic conditions and has been associated with morbidity and mortality.^46^–^49^ Consequently, decreased social functioning in NASH could negatively impact patient quality of life and possibly their prognosis, which highlights the importance of recognizing the severity of this impact when reported by patients and providing the necessary support.

It is important to understand the complete patient experience of NASH, not just its impact on the liver, so that appropriate PRO instruments can be developed. These instruments may then be used to better understand the impact NASH has on patients and could ultimately be used to determine treatment endpoints in clinical trials that may be highly relevant to the patient, despite appearing to be separate from traditional liver disease endpoints. The NASH-CHECK was created by the LITMUS group, whose main aim is to develop and validate biomarkers that achieve a regulatory qualification and can be used for diagnosing and/or monitoring the progression of NAFLD/NASH and fibrosis stage.^50^ The NASH-CHECK was developed to reveal key symptoms and impacts that affect patients with NASH for use in clinical settings, including clinical trials so that the patient-perceived effects of NASH fibrosis stages F1–F3 can be evaluated.^14^ Minor differences were identified between the conceptual model developed in this study and the one created by the LITMUS group.^14^ Although it is unclear why these differences arose, they could have been influenced by patients reporting concepts related to their comorbidities. Further to that, the content included in the conceptual model and NASH-CHECK created by the LITMUS group was reviewed by clinical experts who could have decided to exclude some concepts identified in this study. Overall, this study supports the relevance of the NASH-CHECK findings, demonstrating that this qualitative instrument could help evaluate the holistic impact of NASH, including the impact of NASH on patients’ lives.

Patient interviews to develop the NASH-CHECK^14^ also recorded signs/symptoms and impacts reported most frequently by patients that were similar to those identified in this study, including discomfort in the upper right quadrant, tiredness, cognitive impairment, diet restrictions, and ability to participate in activities. Although reported by Doward et al.^14^, anxiety and worry were reported more frequently by patients in this study. Differences in the saliency of concepts, such as shortness of breath, nausea, abdominal discomfort, daytime sleepiness/feeling drowsy, difficulty thinking clearly, and frustration with medication/health care could have been driven by the presence of patients with cirrhosis (fibrosis stage F4), who were not included in the initial interviews for the NASH-CHECK, and a higher proportion of obese patients. However, overall the studies reported similar experiences for patients; this supports the use of the NASH-CHECK to capture the patient experience of NASH fibrosis stages F1–F3 and that the instrument could also be used for patients with cirrhosis.

### Limitations

The study was restricted in the geographic locations of the patients who participated, and the majority of patients were White non-Hispanic, limiting the ethnic diversity of the study. The small size of the subgroups makes it difficult to make any firm conclusions about the characteristics of and differences between each fibrosis stage, and risks data being skewed by a single patient. A considerable number of symptoms and impacts were elicited only via probing by the interviewer; while we acknowledge this as a limitation, given the nature of NASH and the lack of knowledge in both patients and the general medical community around patient experience of the condition, we are cautious in drawing any inferences regarding the importance/salience of the symptoms/impacts that had to be probed. Over half of patients had diabetes and/or obesity, which could have contributed to some of the concepts being reported as salient. A lack of understanding about NASH could have caused patients to struggle to differentiate between concepts related to NASH and to their comorbid conditions. However, including patients with such comorbidities benefits the study as they are typical conditions experienced by patients with NASH. The study was also conducted during the COVID-19 pandemic so reporting of concepts may have been affected by the encouragement of these individuals to self-isolate (worsening impacts related to self-isolating and patients being more aware of their symptoms). However, a heightened awareness of symptoms could allow some that would otherwise be unrecognized to be identified, allowing a more complete understanding of the condition.

## CONCLUSIONS

This study highlights that patients can self-report and elaborate on signs/symptoms and impacts associated with NASH, regardless of fibrosis stage. However, due to the small sample size, further studies are required to confirm whether this is consistent across patients with NASH, including those from different countries. By identifying concepts that required updating, or were not included in the preliminary conceptual model, the study demonstrates the benefit of talking directly to patients about their experience of NASH. While comorbid conditions, such as obesity and diabetes, may influence the signs/symptoms and impacts reported by patients, these are still important to recognize as part of the patient experience of NASH. The identification of items in the NASH-CHECK that predominantly overlapped with the concepts from this study demonstrates the suitability of the NASH-CHECK for holistically understanding the patient experience of NASH fibrosis stages F1–F4.

## Supplementary Material
