# Supplementary material for: A qualitative patient interview study to understand the experience of patients with nonalcoholic steatohepatitis
Source: Hepatol Commun. 2023 Feb 9;7(3):e0036. doi: 10.1097/HC9.0000000000000036 (PMC9915959; doi:10.1097/HC9.0000000000000036)
Supplement: Supplementary file 1 [file hc9-7-e0036-s001.docx]

**Supporting information**

**SUPPORTING TABLE S1. PUBMED SEARCH TERMS**

| **Category** | **Search terms** |
| --- | --- |
| Disease | “Nonalcoholic steatohepatitis” [Title/Abstract] OR “NASH” [Title/Abstract] OR “Nonalcoholic fatty liver disease” [Title/Abstract] OR “NAFLD” [Title/Abstract] |
| Outcomes of interest | “Signs” OR “Symptoms”[Title/Abstract] OR “Clinical manifestations”[Title/Abstract] OR “Patient reported outcomes”[Title/Abstract] OR “PROs”[Title/Abstract] OR “QoL”[Title/Abstract] OR “HRQoL”[Title/Abstract] OR “HRQL”[Title/Abstract] OR “Quality of life”[Title/Abstract] OR “Conceptual model”[Title/Abstract] OR “Disease model”[Title/Abstract] OR “Qualitative"[Title/Abstract] OR “Focus group"[Title/Abstract] OR “Focus groups”[Title/Abstract] OR “Patient interviews”[Title/Abstract] |

**SUPPORTING TABLE S2. EXCLUSION CRITERIA FOR ARTICLE ABSTRACTS**

| **Source** | **Exclusion criteria** |
| --- | --- |
| Article abstracts | Not related to patient quality of life and not descriptive of signs/symptoms/potential impacts of disease |
|  | Animal studies |
|  | 'Published prior to April 2 2009' |
|  | Considered to be outdated, given rapid evolution of NASH/NAFLD knowledge |

Abbreviations: NFALD, nonalcoholic fatty liver disease; NASH, nonalcoholic steatohepatitis

**SUPPORTING TABLE S3. Eligibility criteria for qualitative patient interviews**

| **NASH fibrosis stage** | **Inclusion** | **Exclusion** |
| --- | --- | --- |
| F1–F3 | Age > 18 years  Had a liver biopsy showing NASH with fibrosis stage F1–F3 ≤ 12 months ago  Stable treatments that could affect NASH (≥12 months of treatment before biopsy) in terms of medication and dose prior to liver biopsy or have not been on a treatment. Including marketed drugs, such as TZDs, GLP-1 analogs and high dose vitamin E (800 IU/d) as well as any investigational drugs tested for the effect on NASH.  Patient resides in any state in the US or Canada (Puerto Rico is excluded) | Fibrosis stage F4  Patient’s body weight has changed by ≥5% in the last 12 months  Patient has started treatment with medication that may affect NASH after the liver biopsy was performed  Other liver diseases  Any other significant medical condition that was recently diagnosed or not stable within the last 3 months and that in the opinion of the investigator makes participation in the study against the patient’s best interests |
| F4 | Age > 18 years  Confirmed NASH fibrosis stage F4 either by biopsy or diagnosed with NASH cirrhosis based on clinical assessment using non-invasive tests/imaging and exclusion of other causes of cirrhosis  Patient resides in any state in the USA or Canada (Puerto Rico is excluded) | Fibrosis stage F1–F3, without clinical evidence of subsequent disease progression to cirrhosis  Patient’s body weight has changed by ≥15% in the last 12 months  Decompensated cirrhosis/F4  Evidence of hepatocellular carcinoma  Other liver disease  Any other significant medical condition that was recently diagnosed or not stable within the last 3 months and that in the opinion of the investigator makes participation in the study against the patient’s best interests  Evidence for hepatic decompensation within the last 3 months |

Abbreviations: GLP-1, glucagon-like peptide 1; NASH, nonalcoholic steatohepatitis; TZD, thiazolidinedione.

**SUPPORTING TABLE S4. TREATMENTS RECEIVED BY PATIENTS FOR COMORBID CONDITIONS**

| **Fibrosis stage** | **Medication** | **N (%)**  **(N = 22)** |
| --- | --- | --- |
| **F1-F3** | Ciclesonide | 1 (4.5) |
|  | Amlodipine | 2 (9.1) |
|  | Hydrochlorothiazide | 1 (4.5) |
|  | Atorvstatin | 1 (4.5) |
|  | Bisopolol | 1 (4.5) |
|  | Clopidogrel | 1 (4.5) |
|  | Naltrexone/Bupropion | 1 (4.5) |
|  | Cyclobenzaprine | 2 (9.1) |
|  | Fluorxetine | 1 (4.5) |
|  | Folic acid | 1 (4.5) |
|  | Dapagliflozin | 1 (4.5) |
|  | Adalimumab | 1 (4.5) |
|  | Irbesartan | 2 (9.1) |
|  | Isosorbide mononitrate | 1 (4.5) |
|  | Empagliflozin | 1 (4.5) |
|  | Sitagliptin/Metformin | 1 (4.5) |
|  | Acetaminophen, codeine, and caffeine | 1 (4.5) |
|  | Metformin | 4 (18.2) |
|  | Mint-gliclazide | 1 (4.5) |
|  | Mirtazapine | 1 (4.5) |
|  | Mylan-fluconazole | 1 (4.5) |
|  | Esomeprazole | 1 (4.5) |
|  | Glyceryl trinitrate | 1 (4.5) |
|  | Olmesartan/ hydrochlorothiazide | 1 (4.5) |
|  | Ozempic | 2 (9.1) |
|  | Rosuvstatin | 1 (4.5) |
|  | Sandox duloxetine alendronate | 1 (4.5) |
|  | Sandoz lansoprazole | 1 (4.5) |
|  | Sandoz Olmesartan | 1 (4.5) |
|  | Levothyroxine | 1 (4.5) |
|  | Telepram | 1 (4.5) |
|  | Acetaminophen/codeine | 1 (4.5) |
|  | Insulin degludec | 1 (4.5) |
|  | Venlafaxine | 1 (4.5) |
|  | Vitamin B12 | 1 (4.5) |
| **F4** | Fluticasone propionate/ salmeterol | 1 (4.5) |
|  | Ibuprofen | 1 (4.5) |
|  | Amoxicillin | 1 (4.5) |
|  | Atorvastatin | 1 (4.5) |
|  | Aspirin | 1 (4.5) |
|  | Tadalafil | 1 (4.5) |
|  | Perindopril | 1 (4.5) |
|  | Rosuvastatin | 2 (9.1) |
|  | Dapagliflozin | 1 (4.5) |
|  | Gliclazide | 1 (4.5) |
|  | Oral glycemics | 1 (4.5) |
|  | Canagliflozin | 1 (4.5) |
|  | Sitagliptin | 1 (4.5) |
|  | Lectulose | 1 (4.5) |
|  | Omeprazole | 1 (4.5) |
|  | Canagliflozin | 1 (4.5) |
|  | Sitagliptin/metformin | 1 (4.5) |
|  | Metformin | 2 (9.1) |
|  | Montelukast | 1 (4.5) |
|  | Pantoprazole | 1 (4.5) |
|  | Perindopril/Indapamide | 1 (4.5) |
|  | PMS-Trazodone | 1 (4.5) |
|  | Ramipril/hydrochlorothiazide | 1 (4.5) |
|  | Repaglinide | 1 (4.5) |
|  | Liraglutide | 1 (4.5) |
|  | Salbutamol HFA | 1 (4.5) |
|  | Mesalazine | 1 (4.5) |
|  | Thiamine | 1 (4.5) |
|  | Acetaminophen | 1 (4.5) |
|  | Salbutamol | 1 (4.5) |
|  | Vitamin D | 1 (4.5) |

**SUPPORTING TABLE S5. PUBLISHED STUDIES IDENTIFIED AS HIGHLY RELEVANT IN THE SYSTEMATIC REVIEW**

|  | **Author** | **Year** | **Title** |
| --- | --- | --- | --- |
| **Article abstract** | Balp et al. | 2017 | Development of a new patient-reported outcome measure for non-alcoholic steatohepatitis: NASH-check |
|  | Cook et al. | 2019 | The Patient Perspectives on Future Therapeutic Options in NASH and Patient Needs |
|  | Doward et al. | 2017 | Exploring the patient perceived impact of non-alcoholic steatohepatitis |
|  | Kennedy-Martin et al. | 2018 | Health-related quality of life burden of nonalcoholic steatohepatitis: a robust pragmatic literature review |
|  | Kopec and Burns | 2011 | Nonalcoholic Fatty Liver Disease: A review of the Spectrum of Disease, Diagnosis, and Therapy |
|  | Newton et al. | 2008 | Fatigue in non-alcoholic fatty liver disease (NAFLD) is significant and associates with inactivity and excessive daytime sleepiness but not with liver disease severity or insulin resistance |
|  | Palsgove et al. | 2016 | Development of a Conceptual Framework for Assessing Disease-Specific Patient-Reported Outcomes in Nonalcholic Steatohepatitis |
|  | Younossi and Henry | 2015 | Economic and Quality-of-Life Implications of Non-Alcoholic Fatty Liver Disease |
|  | Younossi et al. | 2017 | A disease-specific quality of life instrument for non-alcoholic fatty liver disease and non-alcoholic steatohepatitis: CLDQ-NAFLD |
|  | Younossi et al. | 2018 | Clinical and Economic Burden of Nonalcoholic Fatty Liver Disease and Nonalcoholic Steatohepatitis |
|  | Younossi | 2018 | Patient-Reported Outcomes and the Economic Effects of Nonalcoholic Fatty Liver Disease and Nonalcoholic Steatohepatitis: The Value Proposition |
|  | Younossi | 2019 | Non-alcoholic fatty liver disease – a global public health perspective |
| **2019 European Association for the Study of the Liver (EASL) conference** | Ruiz-Margain et al. | 2019 | Effect of a multifactorial intervention (non-alcoholic beer, diet and exercise) on endothelial function, nutritional status and quality of life in patients with cirrhosis |
|  | Taru et al. | 2019 | Minimal hepatic encephalopathy: proper diagnosis for better quality of life |
|  | Younossi et al. | 2019 | What are the Predictors of Impairment of Patient-reported Outcomes in Non-alcoholic Steatohepatitis? |
|  | Humaira Ud-Din et al. | 2019 | Systematic review: Patient reported quality of life outcomes in non-alcoholic fatty liver disease: Effect of disease severity and duration |
|  | Younossi et al. | 2019 | Patients with Non-alcoholic steatohepatitis experience severe impairment of health-related quality of life. |
|  | Heintz et al. | 2019 | Liver fibrosis in conjunction with the prosteatotic PNPLA3 variant affects quality of life in patients with NAFLD: prospective liver stiffness-based study |
|  | Kadler et al. | 2019 | Non-cirrhotic patients with non-alcoholic fatty liver disease have impaired quality of life: independently predicted by body mass index, diabetes and liver stiffness. |
|  | Hefner et al. | 2019 | The Impact of Self Perception of Body Image on Management in Patients with NAFLD. |

**SUPPORTING TABLE S6. PATIENT QUOTES REPRESENTING SALIENT CONCEPTS REPORTED MOST FREQUENTLY BY PATIENTS**

|  | **Concept** | **NASH fibrosis stage** | **Patient number** | **Example quotation** |
| --- | --- | --- | --- | --- |
| **Signs/symptoms** | Fatigue/low energy | F2 | UC05 | “There’s times that I’m very tired and my body doesn’t want to move.” |
|  | Pain in abdomen/liver area | F3 | 001-005 | “But I would get some stomach pains at times, I’d say…that’s been a while. That would be on the side my liver would be on, the right-hand side.” |
|  | GI problems/gassy | F1 | UC01 | “It's not really gassiness, but like upset stomach and diarrhea.” |
| **Impacts** | Worry | F1 | UC08 | “And I was worried about it where I was like, oh my god, is my liver destroyed? Am I? So it was a very stressful, very uncertain time.” |
|  | Restricted in the foods eaten | F4 | UC11 | “Well, it’s impacting me as well as my spouse. Every day we’re looking at what we’re eating and trying to make the right choices. So when we go out, it’s a little more difficult.” |
|  | Anxiety | F3 | 001-004 | “I have anxiety, absolutely. When that happens, I feel like I want to jump out of my own skin, but I don’t have the energy. I’m angry, I’m upset, I find myself crying at times.” |
|  | Frustration | F1 | 001-006 | “I would definitely say that just overall the toll it takes on my life is just annoying and frustrating.” |
|  | Decreased ability to do daily activities | F3 | 001-004 | “Never in my life did I think at almost 50 would my life be so restricted and unproductive. I feel like I’m a person in my 80s with not being very active, enjoying life. Making excuses not to go anywhere.” |
|  | Daytime sleepiness / feeling drowsy | F1 | 001-002 | “Just because that sleepiness… Like I said, it will come on, I’ll need to take a nap, I’ll need to stop and just take that time to lay back and put my feet up. Or go upstairs and lay down for a bit.” |
|  | Unhappiness | F2 | 001-009 | “Not angry but what you said, irritable and unhappy. I think it goes in combination with those.” |

Abbreviation: GI, gastrointestinal.

**SUPPLEMENTARY TABLE S7. UPDATES TO PRELIMINARY CONCEPTUAL MODEL FOLLOWING CONCEPT ELICITATION INTERVIEWS**

|  | **Concept** | **Update** |
| --- | --- | --- |
| **Signs/symptoms** | Cognitive problems   - Difficulty thinking clearly - Difficulty following a conversation - Problems focusing - Forgetful | Updated from ‘Cognitive problems’ |
|  | GI problems (constipation, diarrhoea, upset stomach, flatulence) | Updated from ‘GI problems/gassy’ |
|  | Nausea/vomiting | Updated from ‘Nausea’ |
|  | Ascites/ascites leaks | Removed |
|  | Bleeding | Removed |
|  | Clammy palms | Removed |
|  | Metallic taste in mouth | Removed |
| **Impacts** | Decreased ability to do daily activities | Added |
|  | Impact on family/friends | Added |
|  | Restricted in food eaten | Added |
|  |  |  |
|  | Depression/Sadness | Updated from ‘Depression’ |
|  | Difficulty staying asleep | Updated from ‘Poor sleep quality’ |
|  | Frustration   - Frustration with medication/healthcare - Frustration with diet | Combined ‘Frustration’, ‘Frustration with medication/healthcare’ and ‘Frustration with diet’ |
|  | Emotional impact | Removed |
|  | Decreased ability to work | Moved from ‘Immediate impacts’ to ‘General impacts’ |
|  | Worry | Moved from ‘General impacts’ to ‘immediate impacts’ |

Abbreviation: GI, gastrointestinal

**SUPPORTING TABLE S8. CONCEPTS REPORTED BY AT LEAST 20% OF PATIENTS DURING INTERVIEWS COMPARED WITH ITEMS REPORTED IN THE NASH-CHECK**

| **Concepts** | **Measured by NASH-CHECK** |
| --- | --- |
| **Signs/symptoms** | |
| Abdominal bloating | Yes |
| Abdominal discomfort | Yes* |
| Abdominal pressure/tightness | No |
| Bodily pain | No |
| Cognitive problems | No |
| Dermatological issues (redness/psoriasis) | Yes* |
| Dizziness | No |
| Dry mouth | No |
| Fatigue/low energy | Yes |
| Flu-like symptoms | No |
| GI problems/gassy | No |
| Joint pain | No |
| Loss of appetite/feeling full quickly | No |
| Metallic taste in mouth | No |
| Nausea | No |
| Pain in abdomen/liver area | Yes |
| Pruritus/itching | Yes |
| Shortness of breath | No |
| Spider-like veins | No |
| **Impacts** | |
| Anger | Yes |
| Anxiety | Yes* |
| Daytime sleepiness/feeling drowsy | Yes |
| Decreased ability to do daily activities | Yes |
| Decreased ability to do work | Yes |
| Decreased physical activity** | Yes |
| Decreased role functioning | No |
| Decreased social functioning | Yes |
| Depression/sadness | Yes |
| Difficulty following a conversation | Yes |
| Difficultly sleeping | Yes |
| Difficulty thinking clearly | Yes |
| Financial impacts | No |
| Forgetful | Yes |
| Frustration | No |
| Frustration with diet | Yes* |
| Frustration with medication/healthcare | No |
| Impact on family/friends | Yes |
| Impact on self-confidence/self esteem | Yes |
| Irritability | No |
| Problems focusing | Yes |
| Psychological/psychiatric issues | No |
| Restricted in foods eaten | Yes |
| Unhappiness | Yes* |
| Worry | Yes |

Abbreviations: GI, gastrointestinal; NASH, nonalcoholic steatohepatitis; NASH-CHECK, NASH-specific patient-reported outcome instrument.

*Concept identified during the patient interviews was covered by the NASH-CHECK via overlap with another concept

**Includes difficulty carrying heavy objects, doing light chores, doing heavy chores, walking (brisk), walking (long), walking (short), and walking upstairs
